# Supplementary material for: A hyaena on stilts: comparison of the limb morphology of Ictitherium ebu (Mammalia: Hyaenidae) from the Late Miocene of Lothagam, Turkana Basin, Kenya with extant Canidae and Hyaenidae
Source: PeerJ. 2024 Jun 10;12:e17405. doi: 10.7717/peerj.17405 (PMC11172688; doi:10.7717/peerj.17405)
Supplement: Supplemental Information 3 — Most of these specimens did not have the distal humerus correctly aligned with the camera. [file peerj-12-17405-s003.docx]

| Specimen | Species | Reason for exclusion |
| --- | --- | --- |
| RMNH MAM 35105 | *Hyaena hyaena* | Deformed humerus |
| NRM 20135299 | *Canis lupus* | Angle not aligned with camera |
| RMCA 18766 | *Lupulella adusta* | Angle not aligned with camera |
| RMCA 33283 | *Lupulella adusta* | Angle not aligned with camera |
| RMCA 17806 | *Lupulella adusta* | Angle not aligned with camera |
| RMNH MAM 9858 | *Vulpes vulpes* | Angle not aligned with camera |
| ZMB MAM 40121 | *Chrysocyon brachyurus* | Angle not aligned with camera |
