# Supplementary figures and images for: A hyaena on stilts: comparison of the limb morphology of Ictitherium ebu (Mammalia: Hyaenidae) from the Late Miocene of Lothagam, Turkana Basin, Kenya with extant Canidae and Hyaenidae
Source: PeerJ. 2024 Jun 10;12:e17405. doi: 10.7717/peerj.17405 (PMC11172688; doi:10.7717/peerj.17405)

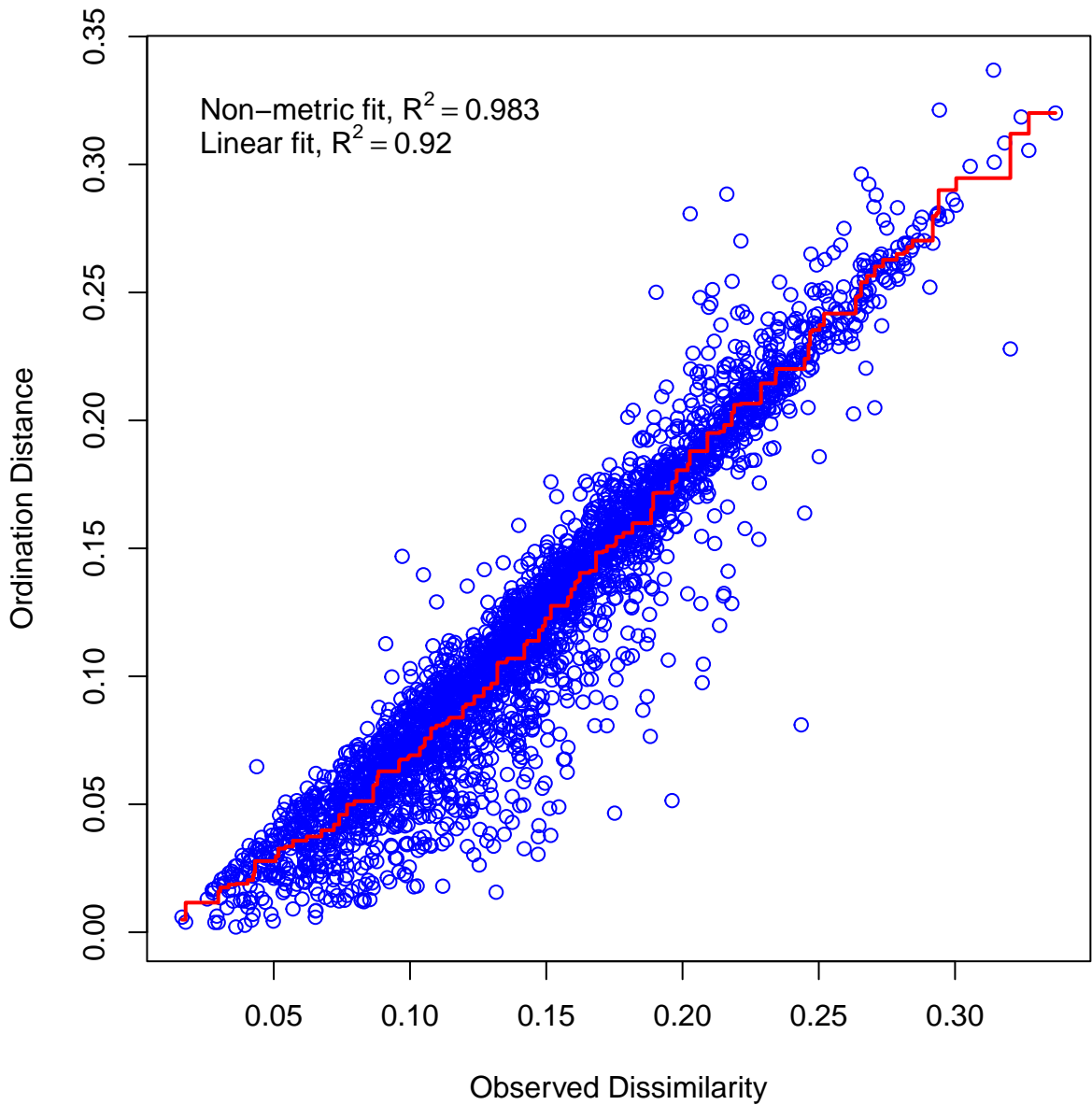

Supplement: Supplemental Information 10 — The x-axis represents the original distances between points in the data. The y-axis represents the distances adjusted by the NMDS. R2 is a measure of the goodness of fit, which is highest at 1. [file peerj-12-17405-s010.pdf]

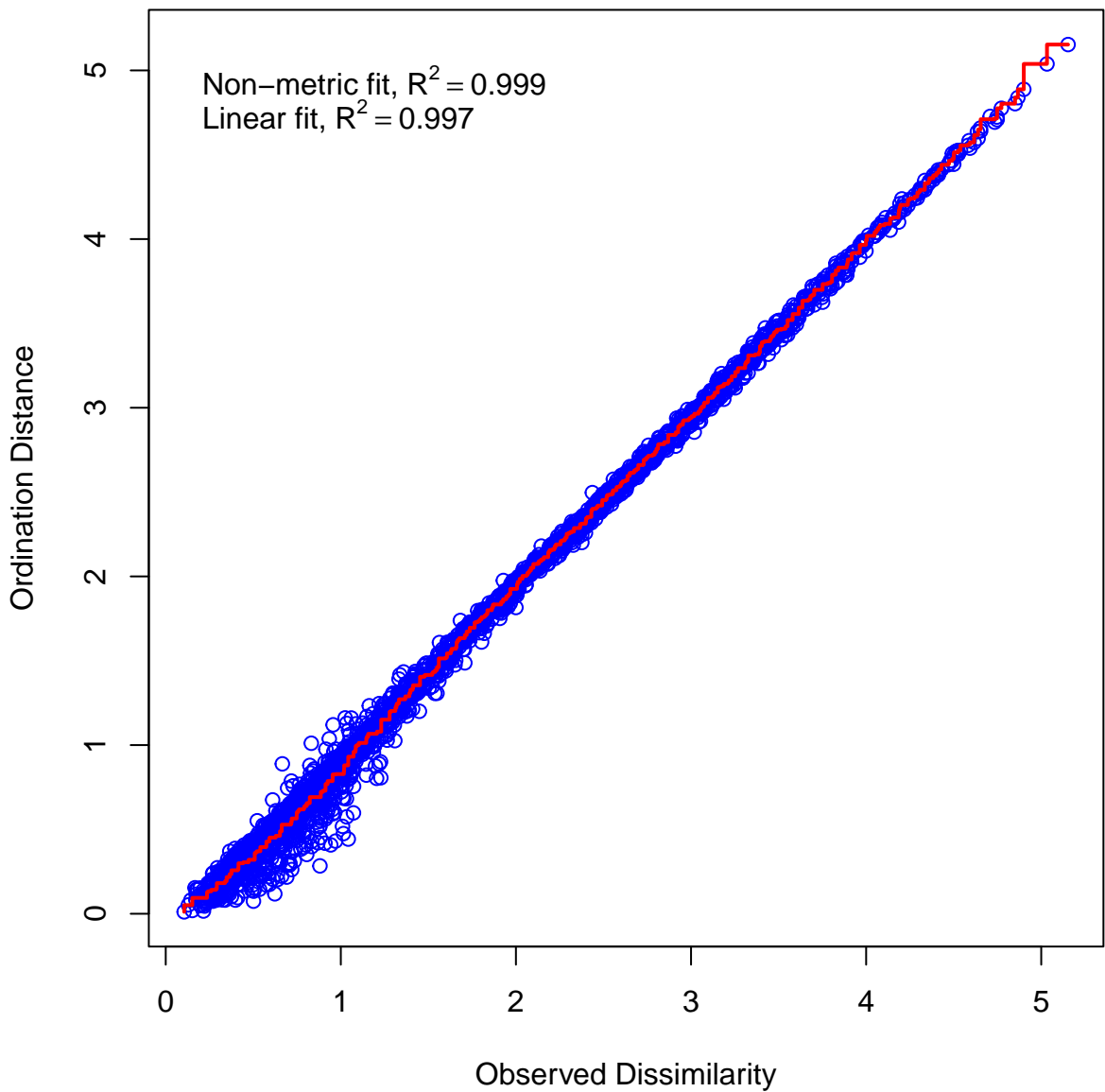

Supplement: Supplemental Information 11 — The x-axis represents the original distances between points in the data. The y-axis represents the distances adjusted by the NMDS. R2 is a measure of the goodness of fit, which is highest at 1. [file peerj-12-17405-s011.pdf]

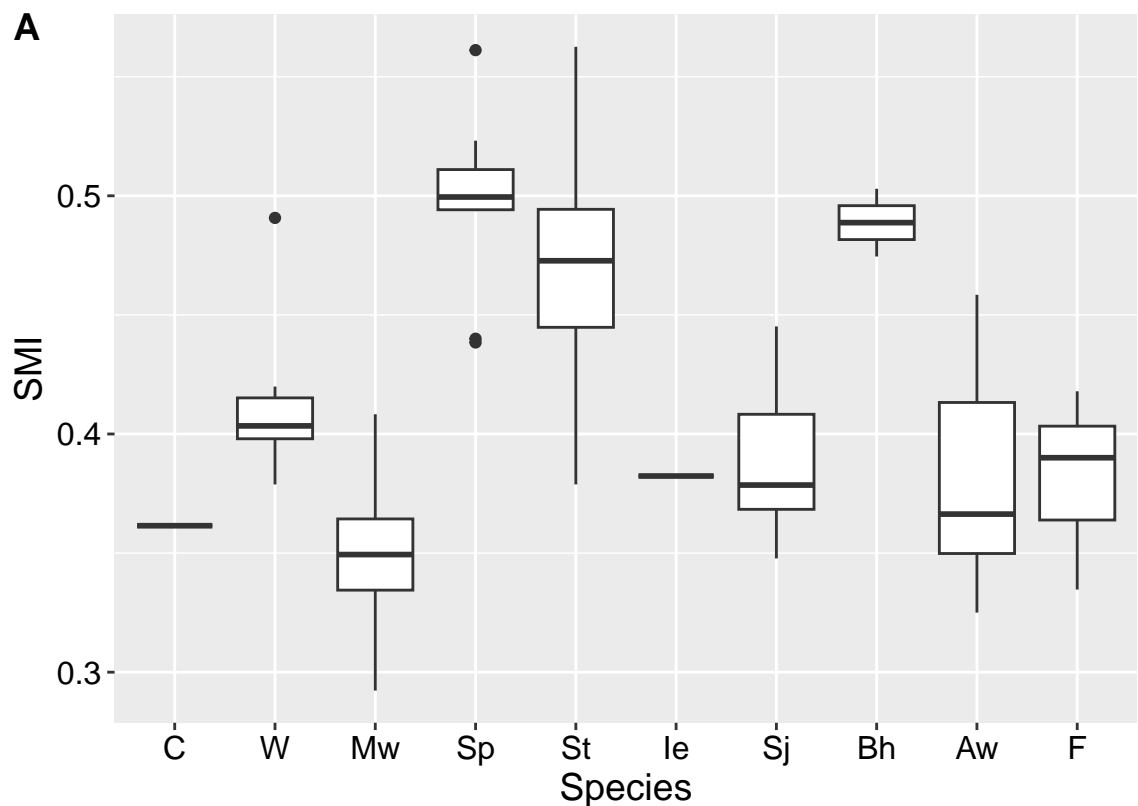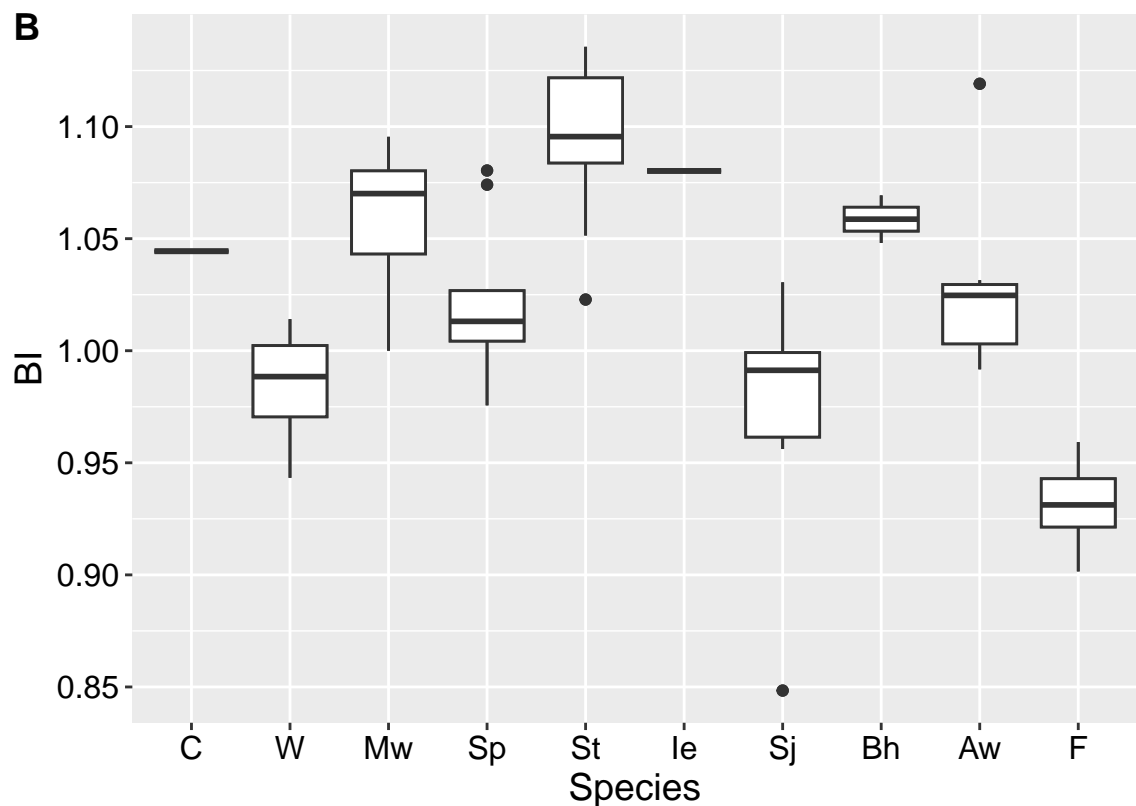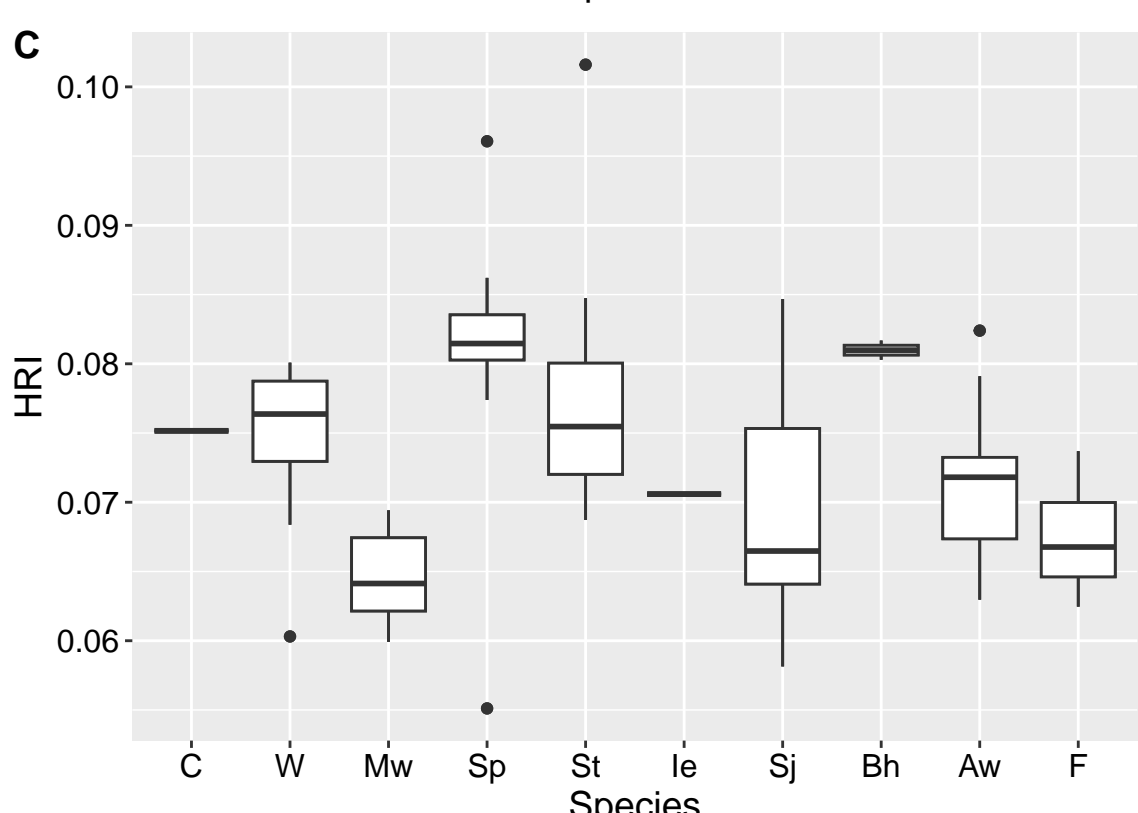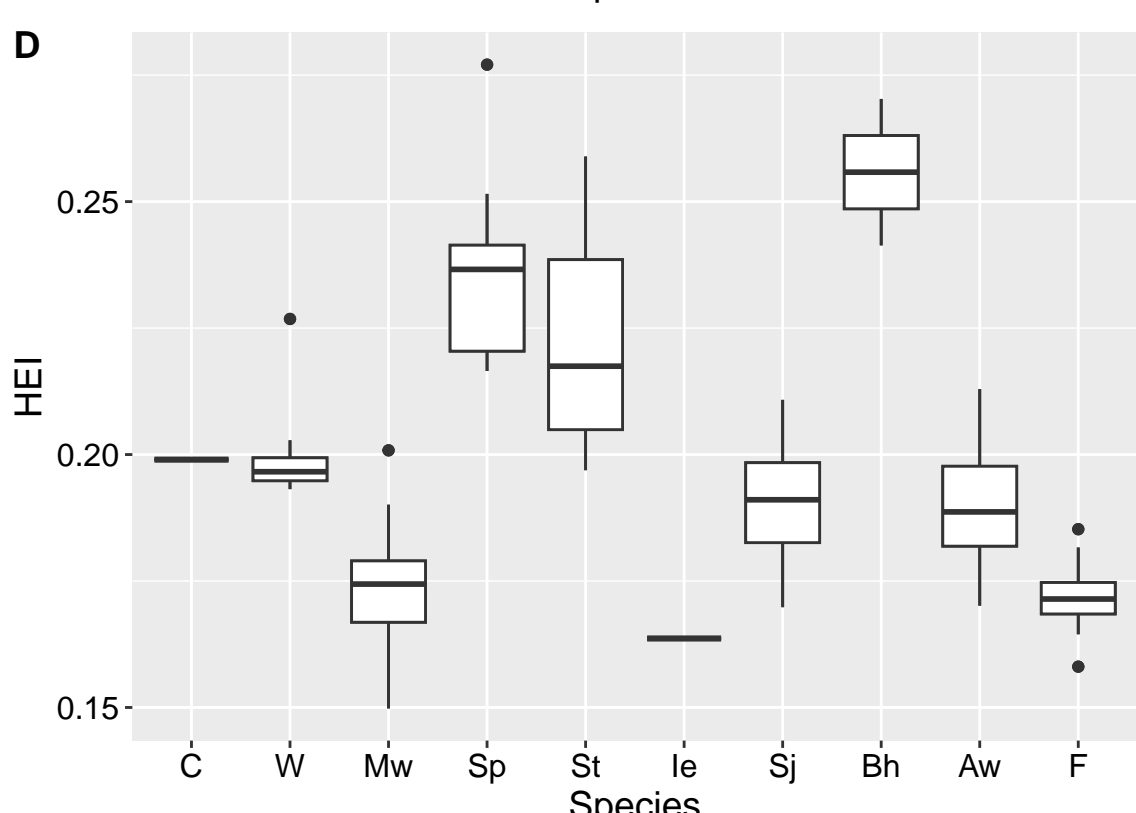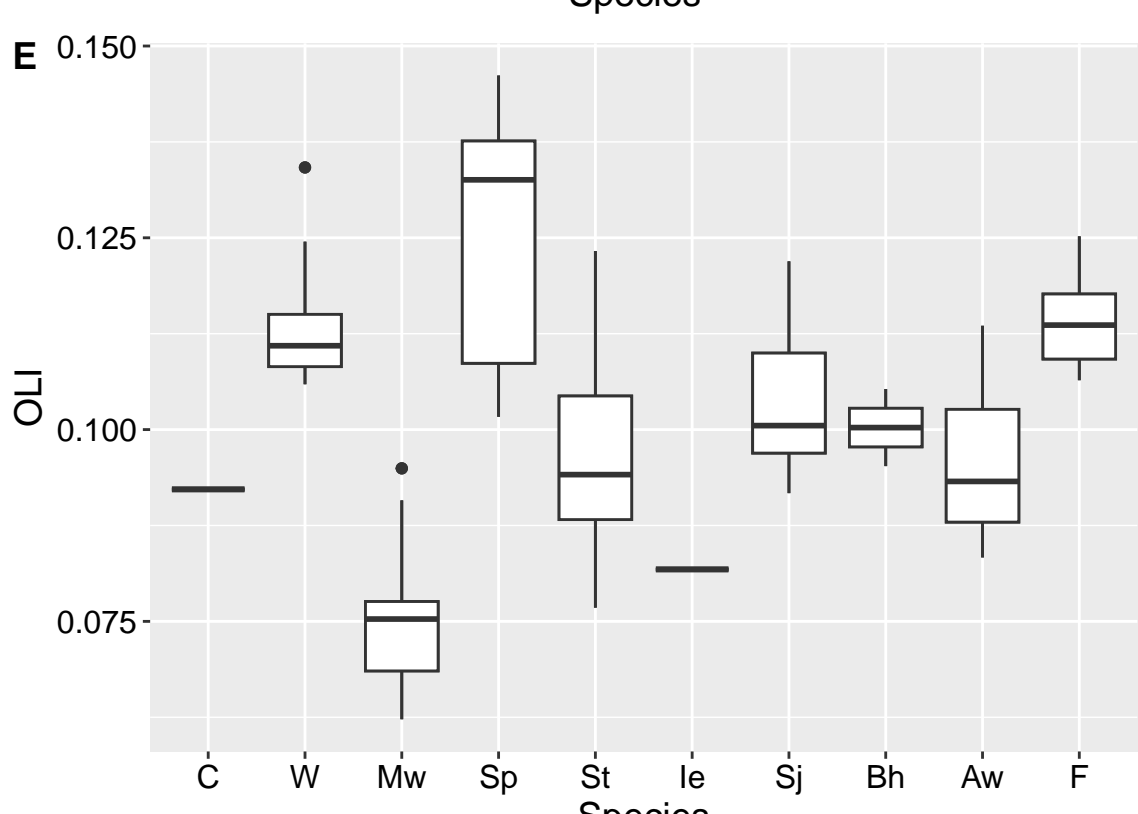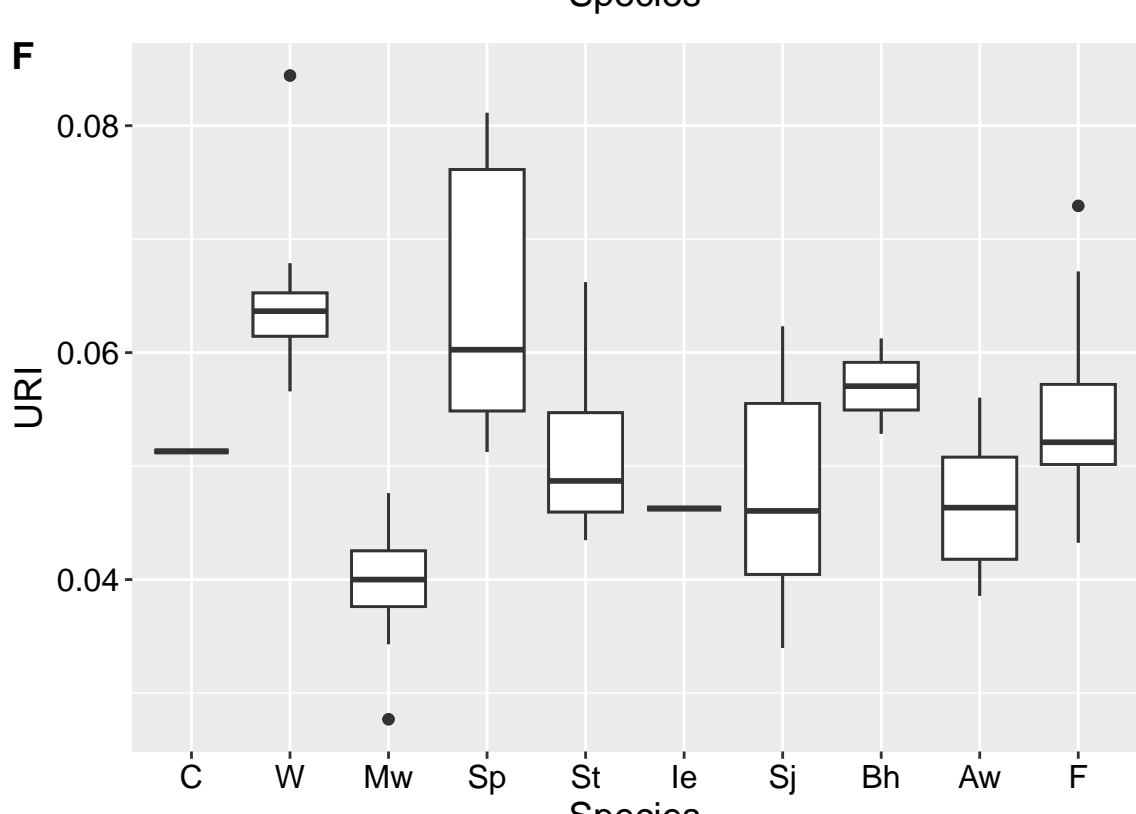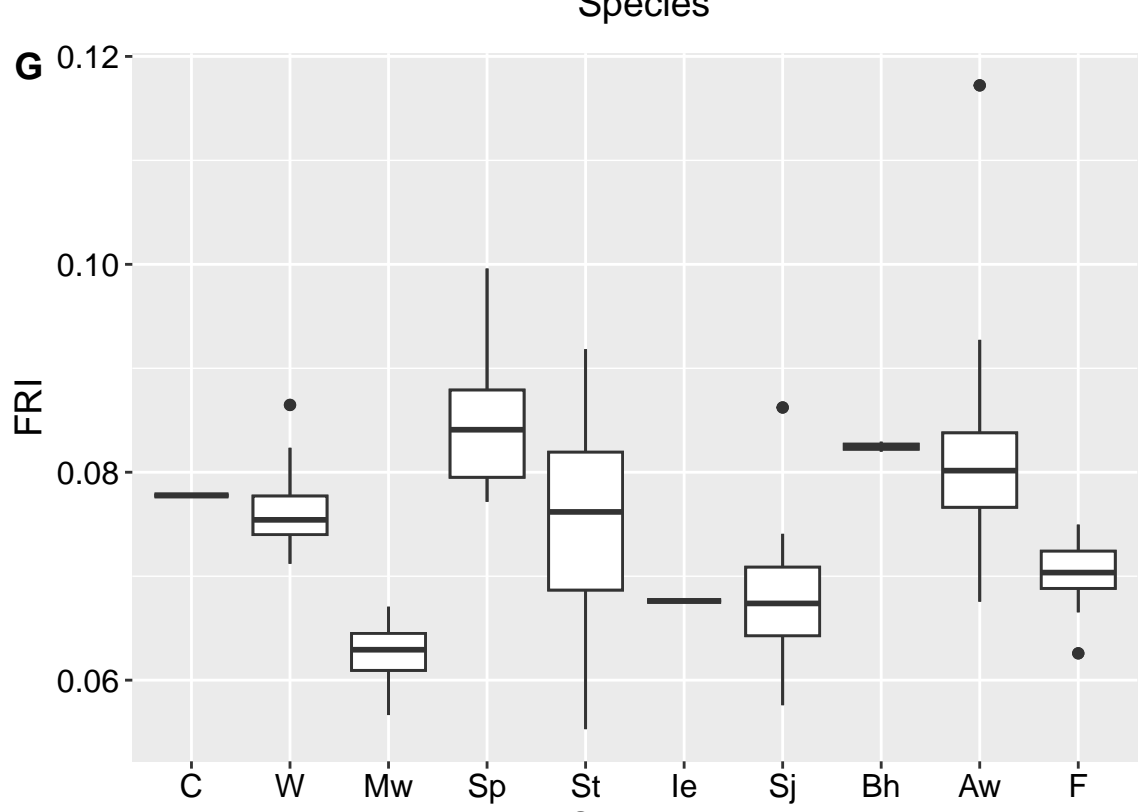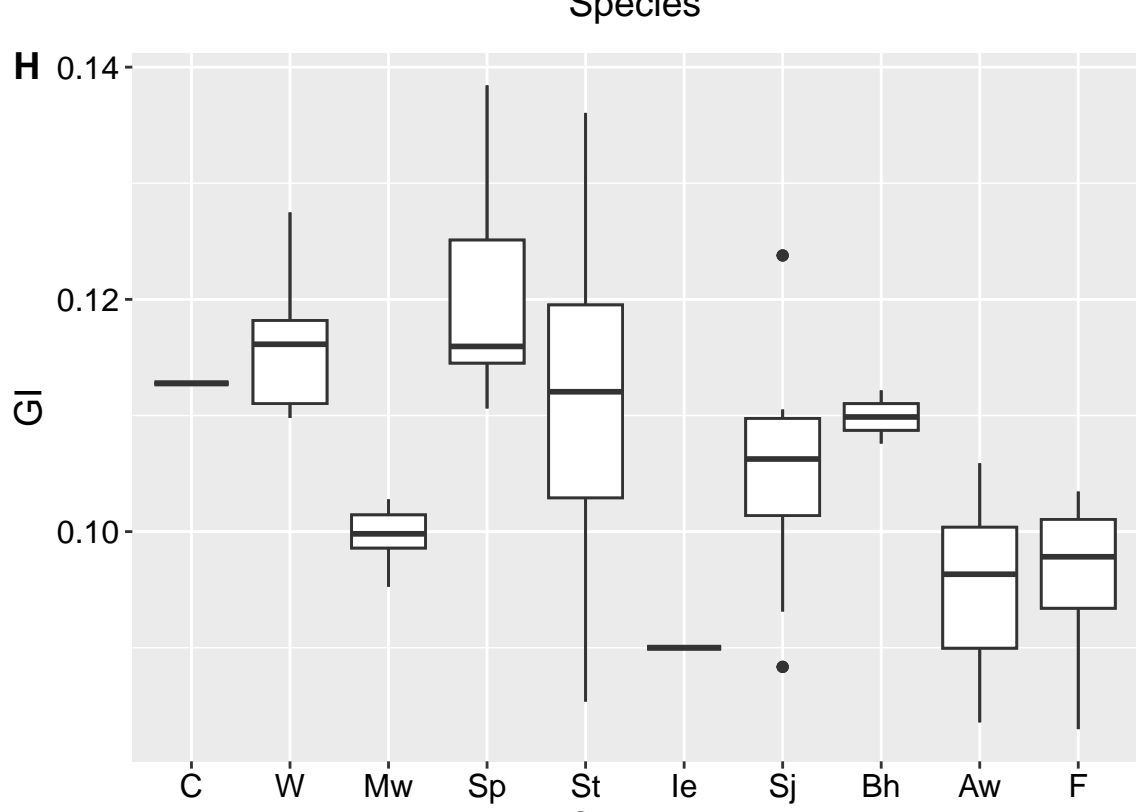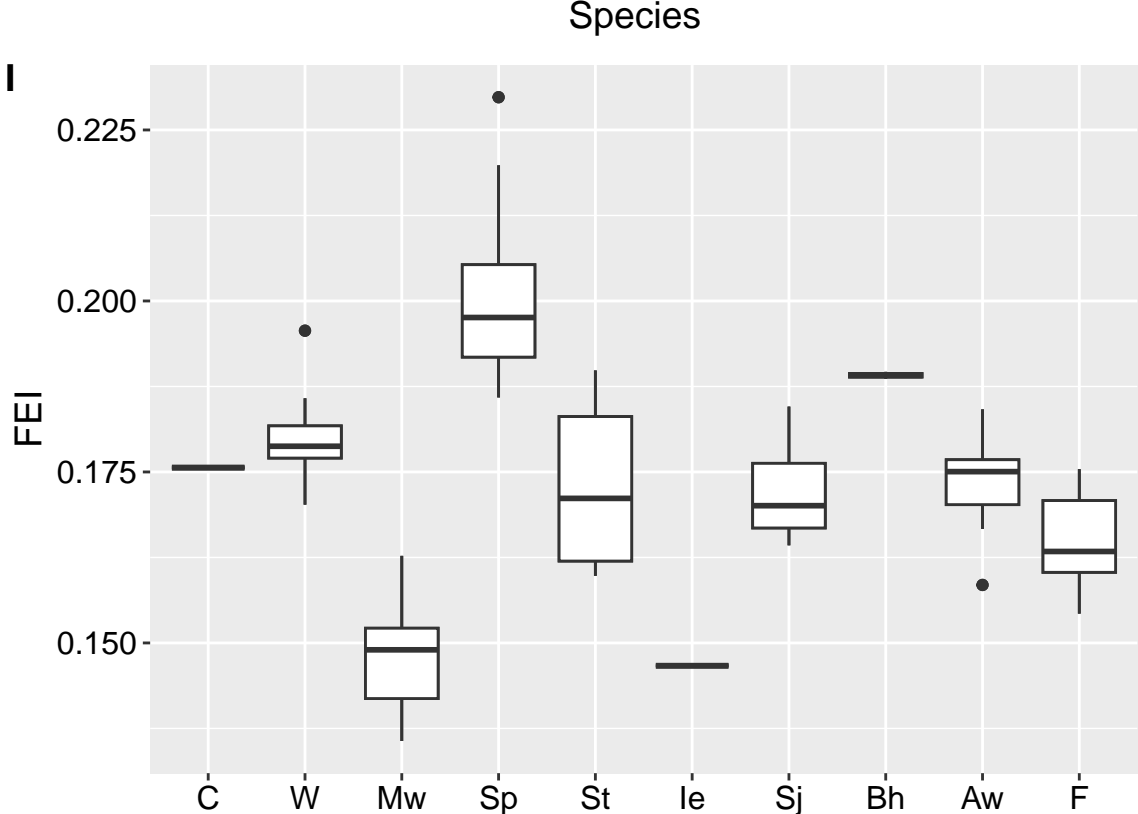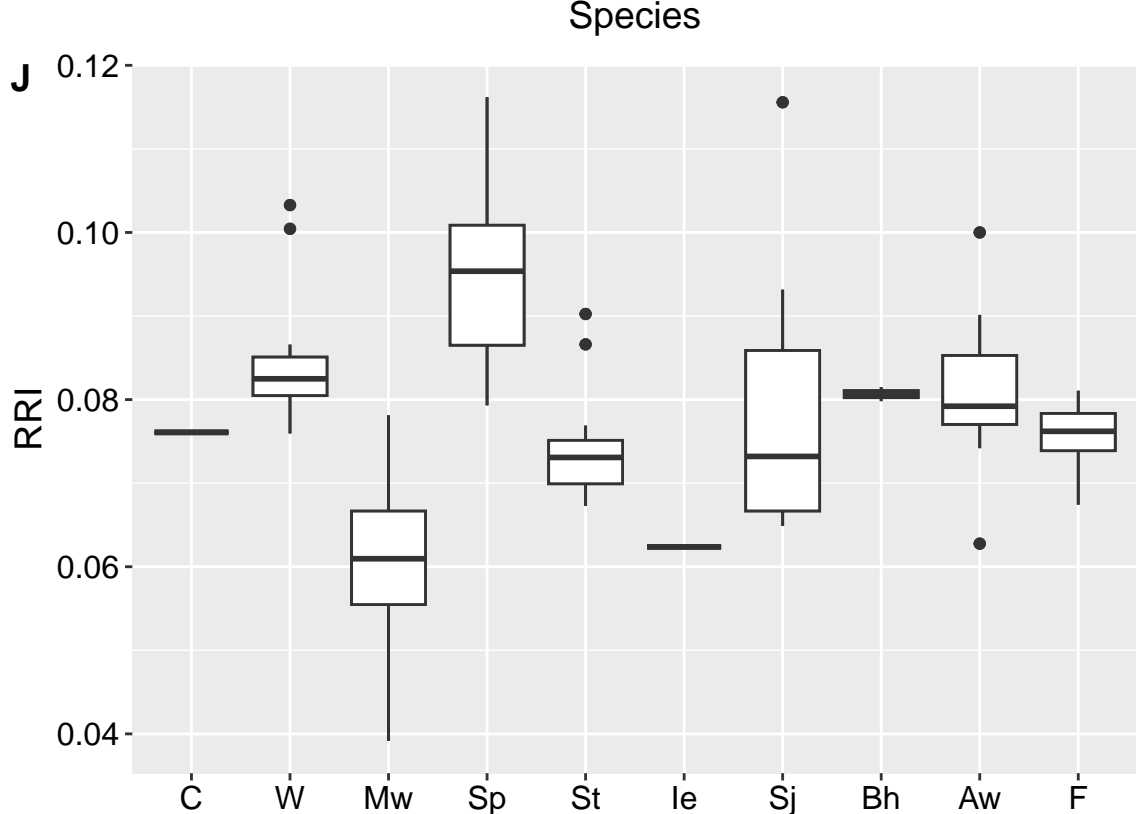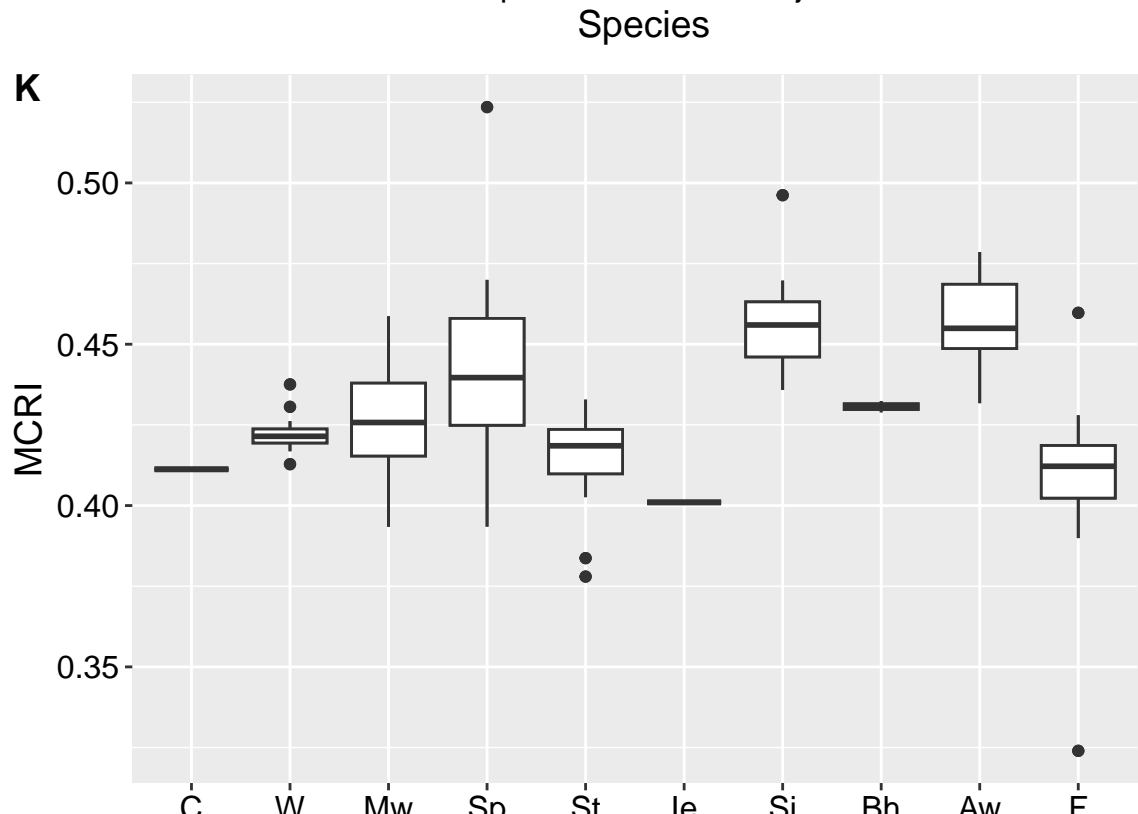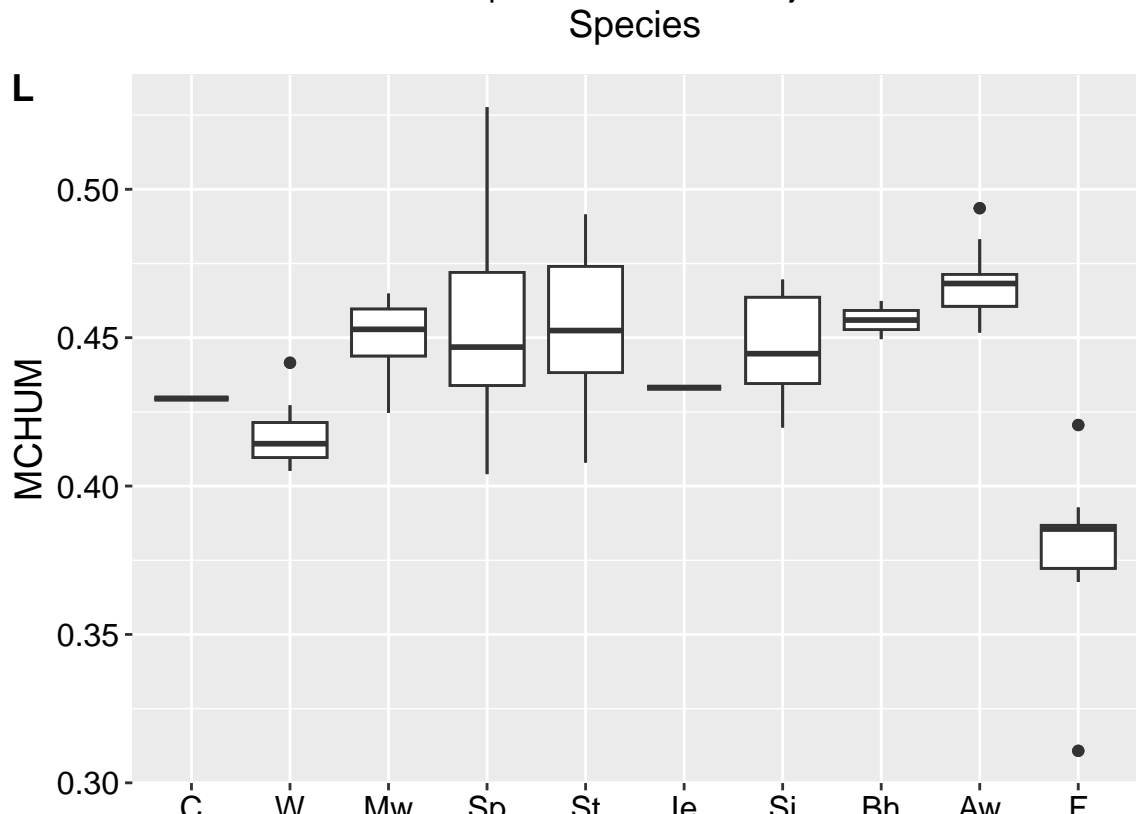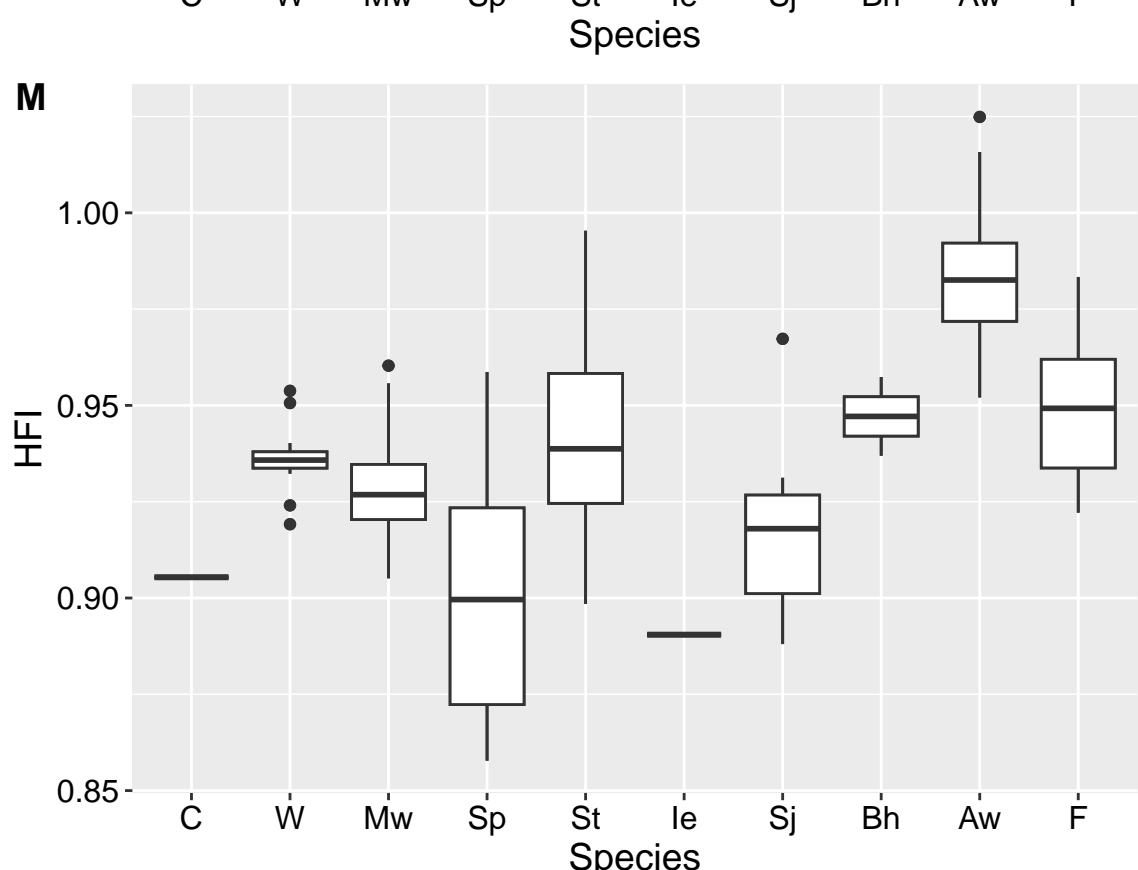

Supplement: Supplemental Information 12 — (A) Shoulder moment index. (B) Branchial index. (C) Humeral robustness index. (D) Humeral epicondylar index. (E) Olecranon length index. (F) Ulnar robustness index. (G) Femoral robustness index. (H) Gluteal index. (I) Femoral epicondylar index. (J) Radial robustness index. (K) Metacarpal radial index. (L) Metacarpal humeral index. (M) Humeral femoral index. C = Coyote, W = Wolf, Mw = Maned wolf, Sp = Spotted Hyaena, St = Striped hyaena, Ie = Ictitherium ebu, Sj = Side-striped jackal, Bh = Brown hyaena, Aw = Aardwolf, F = Fox. [file peerj-12-17405-s012.pdf]

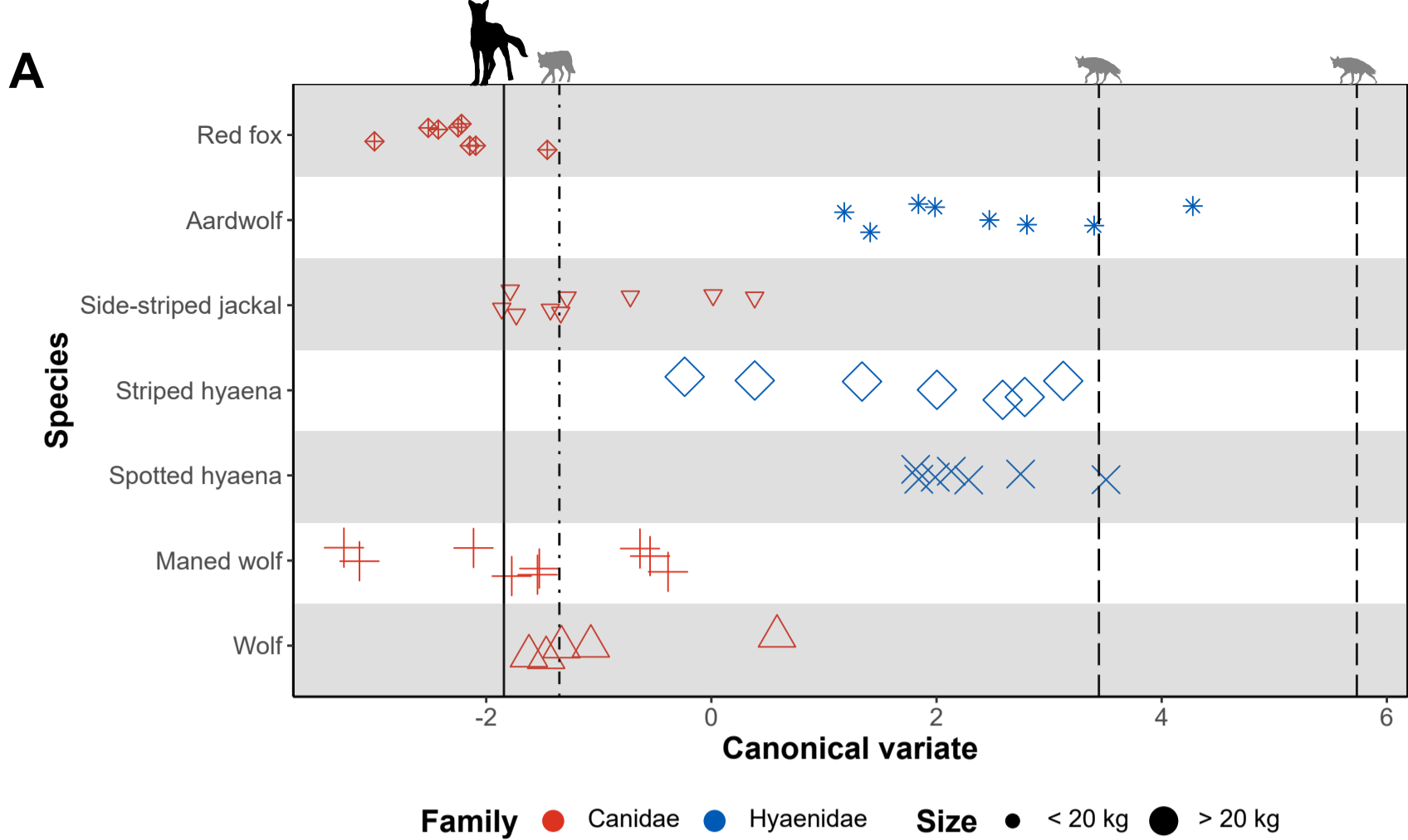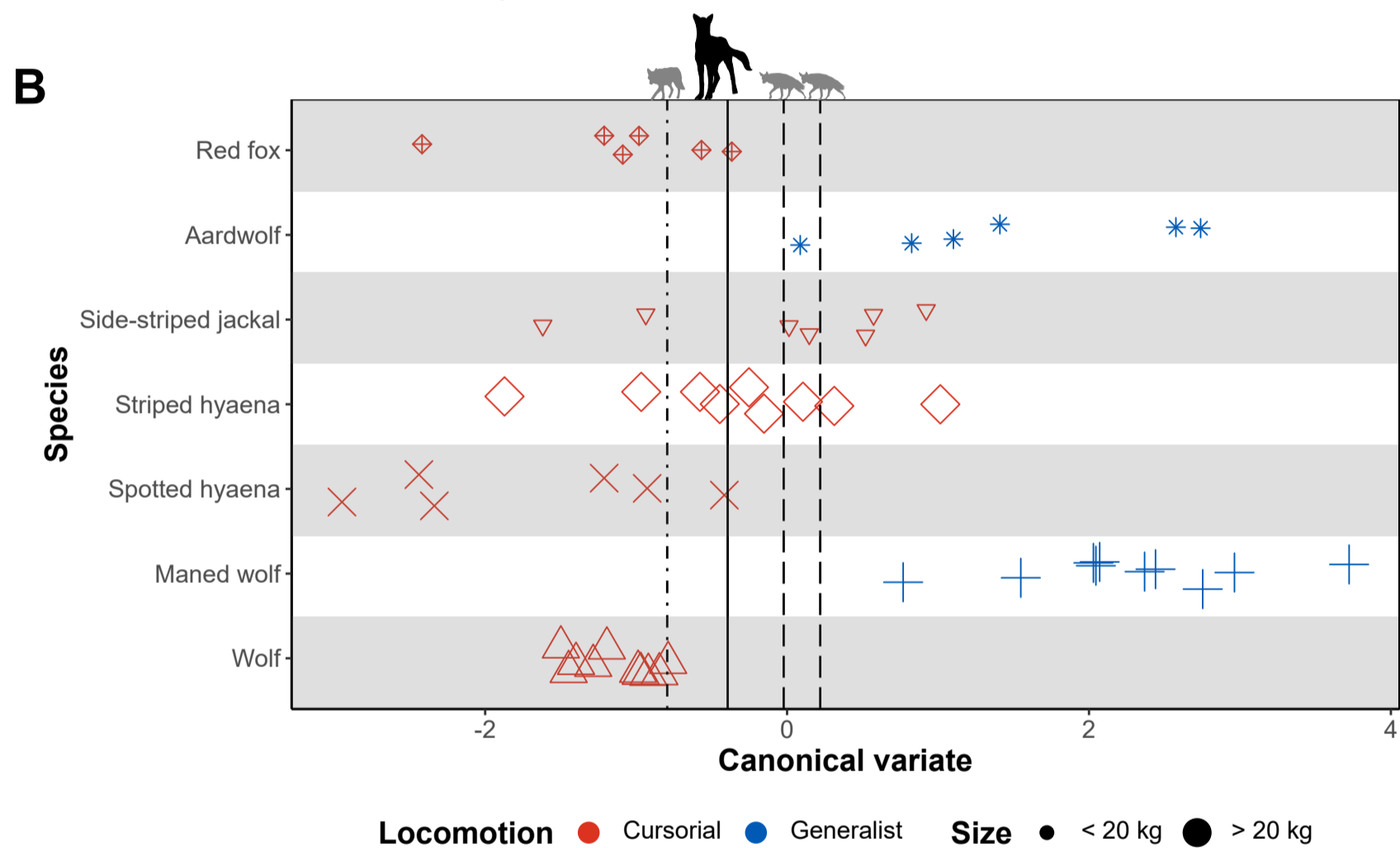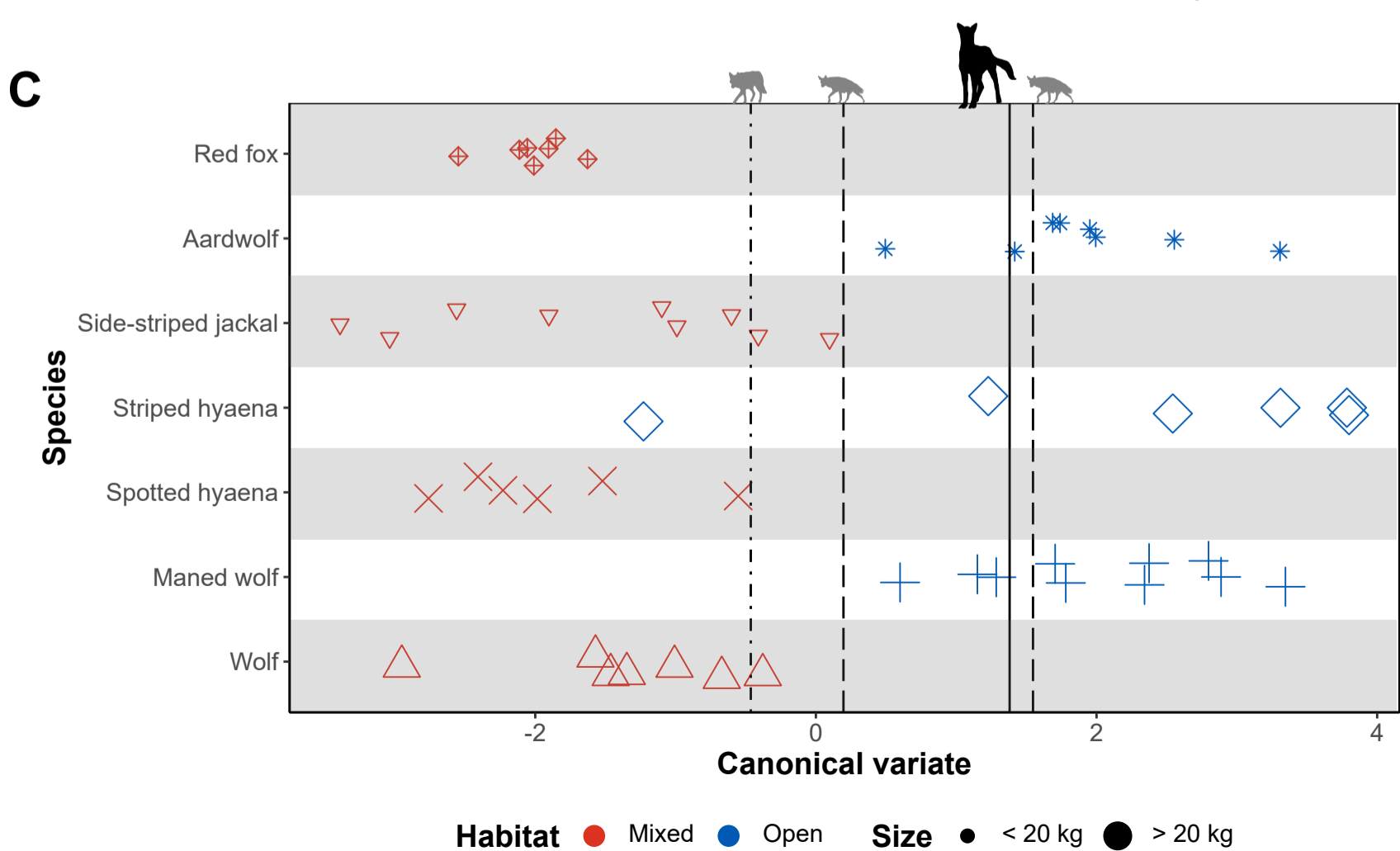

Supplement: Supplemental Information 13 — Points are jittered to allow for a clearer view of the data. Groups are labelled by colour, species by shape. The silhouette of I.ebu is in black. The prediction of I. ebu is plotted as a line. Extant species are in grey. The coyote is plotted as a dot-dash line and the brown hyaenas are plotted as dashed lines. Silhouette of Ictitherium ebu traced from the reconstruction by Javier Herbozo. Extant species silhouettes from Phylopic (Keesey, 2023): Brown hyaena: https://www.phylopic.org/images/55b1ad9b-93d5-4fa9-83a5-4231c07c8620/hyaena-brunnea. Coyote: https://www.phylopic.org/images/5a0398e3-a455-4ca6-ba86-cf3f1b25977a/canis-latrans. [file peerj-12-17405-s013.pdf]

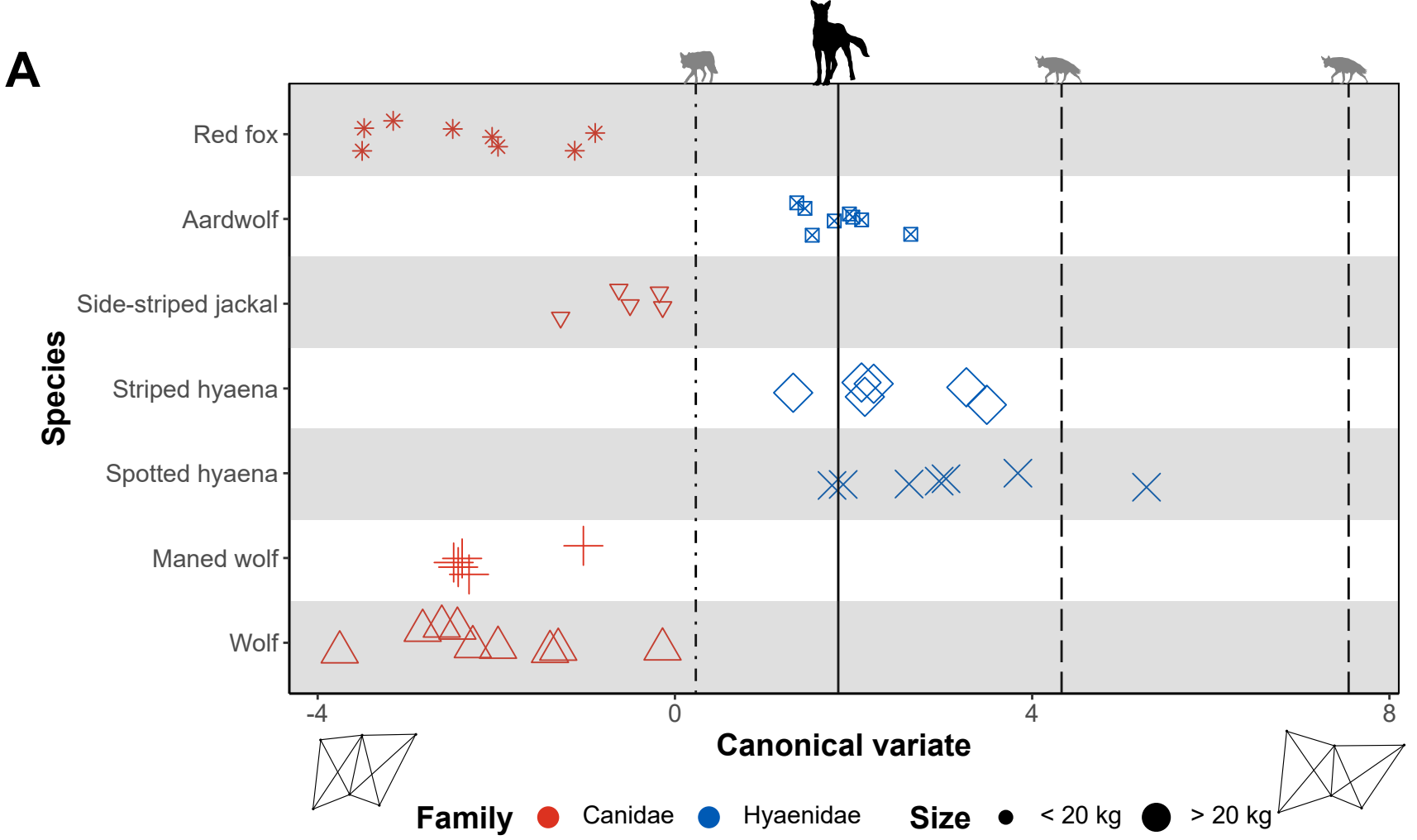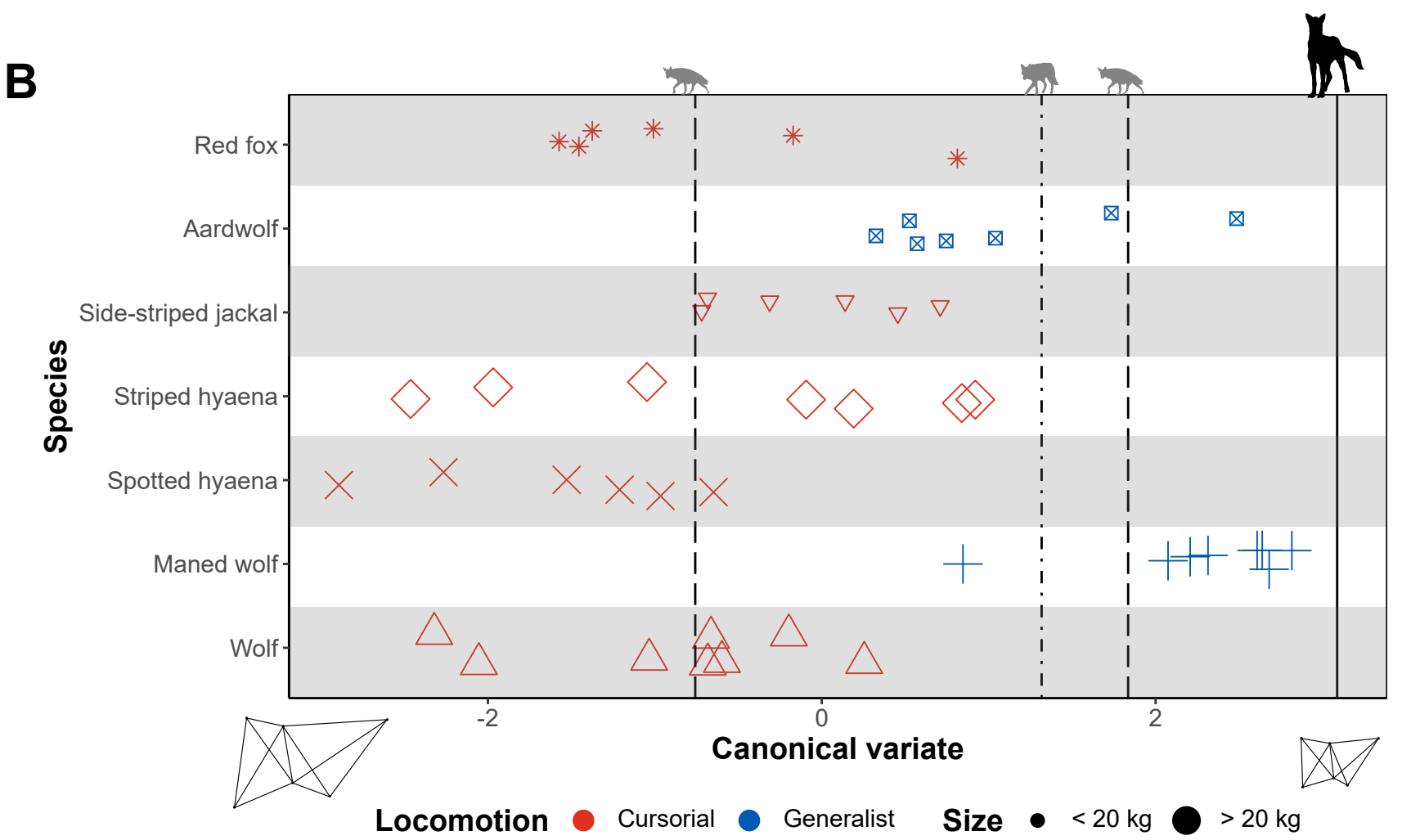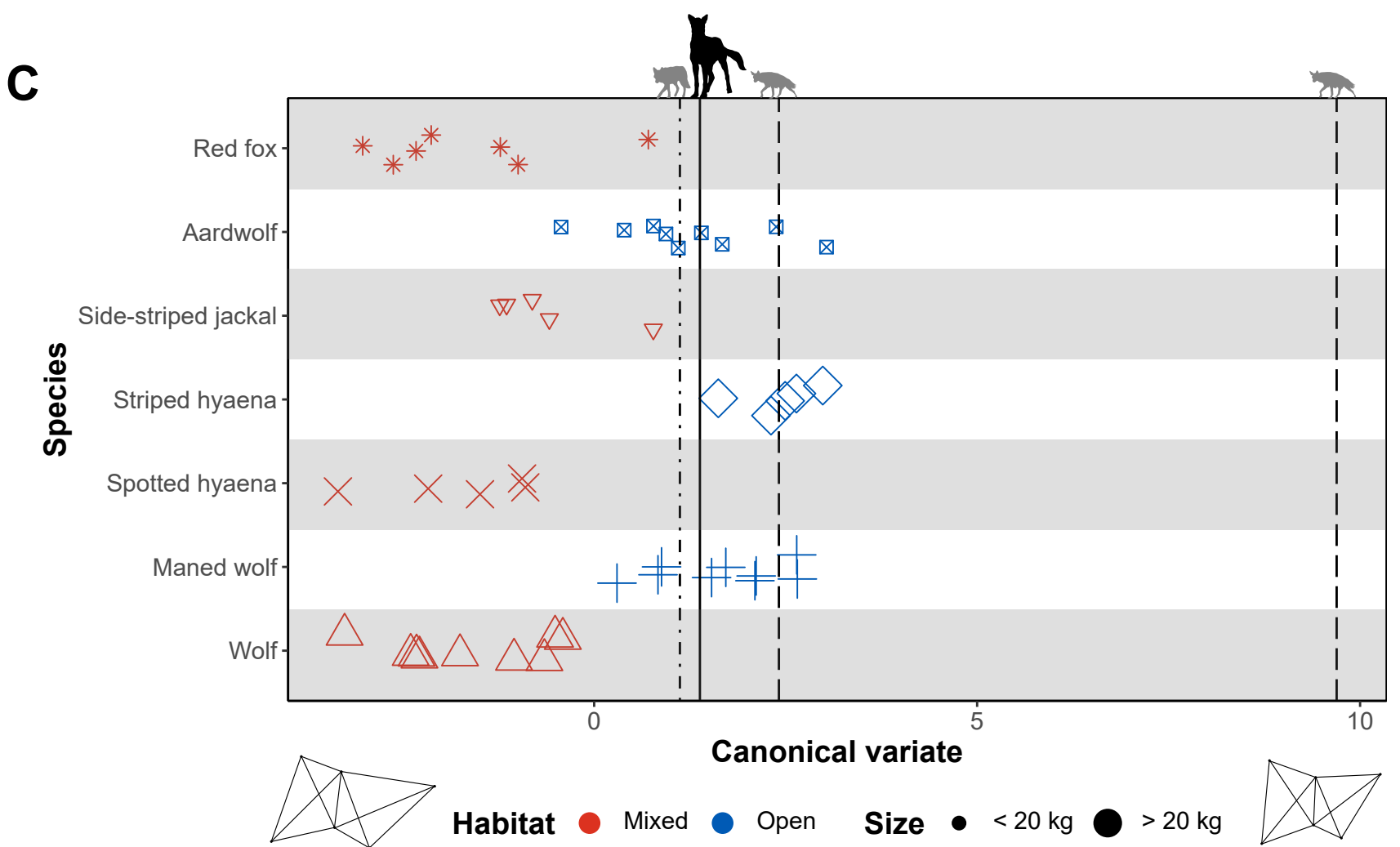

Supplement: Supplemental Information 14 — Points are jittered to allow for a clearer view of the data. Groups are labelled by colour, species by shape. The silhouette of I.ebu is in black. The prediction of I. ebu is plotted as a line. Extant species are in grey. The coyote is plotted as a dot-dash line and the brown hyaenas are plotted as dashed lines. Silhouette of Ictitherium ebu traced from the reconstruction by Javier Herbozo. Extant species silhouettes from Phylopic (Keesey, 2023): Brown hyaena: https://www.phylopic.org/images/55b1ad9b-93d5-4fa9-83a5-4231c07c8620/hyaena-brunnea. Coyote: https://www.phylopic.org/images/5a0398e3-a455-4ca6-ba86-cf3f1b25977a/canis-latrans. [file peerj-12-17405-s014.pdf]

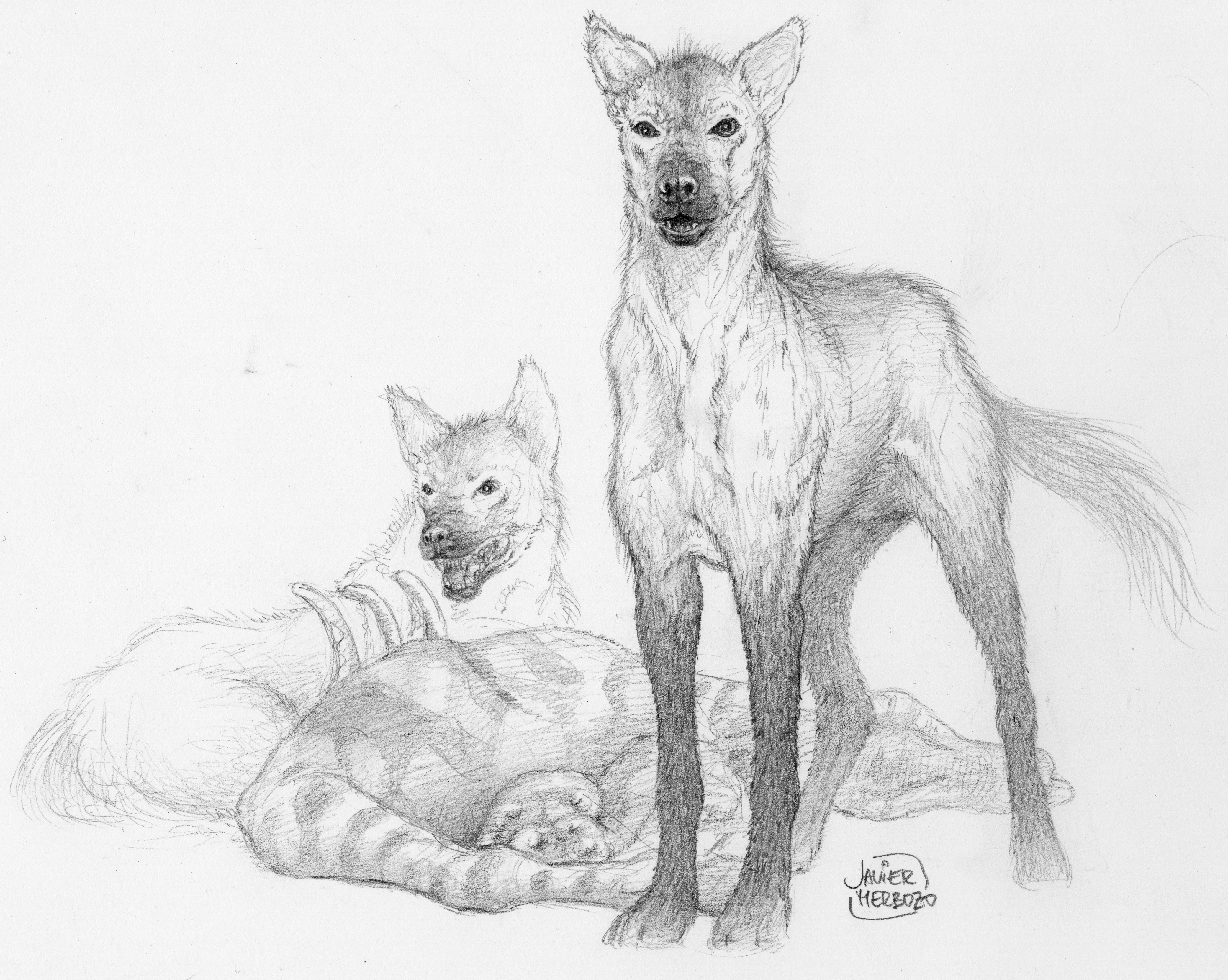

Supplement: Supplemental Information 15 [file peerj-12-17405-s015.png]
